# Inactivation of *Dicer1 *in Steroidogenic factor 1-positive cells reveals tissue-specific requirement for *Dicer1 *in adrenal, testis, and ovary

**DOI:** 10.1186/1471-213X-10-66

**Published:** 2010-06-11

**Authors:** Chen-Che J Huang, Humphrey HC Yao

**Affiliations:** 1 Department of Veterinary Biosciences, University of Illinois, Urbana, IL, USA

## Abstract

**Background:**

The synthesis of microRNA (miRNA) is a multi-step process that requires the action of the ribonuclease Dicer1. Dicer1 is responsible for the final processing of miRNA and has been implicated in cellular processes such as proliferation, apoptosis, and differentiation. Mouse embryos lacking *Dicer1 *die in early embryogenesis. In this study, we investigated whether *Dicer1 *is required for development of adrenal, testis, and ovary in mouse embryos.

**Results:**

To target *Dicer1 *deletion specifically in developing adrenals and gonads, we used Steroidogenic factor 1-cre (*Sf1/Cre*) line in which Cre recombinase is active in the progenitor cells of adrenals and gonads. Lack of *Dicer1 *in the SF1-positive cells did not affect formation and early differentiation of the adrenals and gonads. However, increasing numbers of apoptotic cells were first detected in the *Dicer1 *knockout adrenal cortex at 18.5 days post coitum (dpc), followed by apoptosis of somatic cells and germ cells in the testis at postnatal day 0. Affected adrenal and testes underwent complete degeneration 48 hrs after the onset of apoptosis. However, ovaries were not affected at least until postnatal day 5, when the animals died due to adrenal insufficiency.

**Conclusions:**

*Dicer1 *is dispensable for formation and differentiation of fetal tissues derived from the SF1-positive adrenogonadal primordium. *Dicer1 *is essential for maintaining cell survival in adrenal and testis; however, development of the ovary from fetal stages to postnatal day 5 does not require the presence of *Dicer1*. Our results reveal a tissue-specific requirement of *Dicer1 *and microRNAs. Future research is needed to understand how the tissue-specific role of *Dicer1 *is established.

## Background

*Dicer1 *gene encodes a protein containing RNase III domains essential for miRNA biogenesis. miRNAs, which are 19-25 nucleotides long, non-coding RNAs, regulate gene expression by binding to target mRNAs in a sequence-specific manner, subsequently inhibiting their translation or inducing their degradation [1-3]. This post-transcriptional gene regulation machinery has been implicated in controlling diverse aspects of development in organisms from plants to mammals. In mice, general knockout (KO) of *Dicer1 *resulted in embryonic lethality around 7.5 dpc [4]. Inability of *Dicer1 *KO embryonic stem cells to develop further highlights the role of miRNA machinery in maintaining stem cell population at early developing stages. Results from the tissue-specific KO of *Dicer1 *gene in mice have demonstrated the importance of miRNAs in organogenesis including heart, lung, limb and gonads [5-11].

Adrenal, testis, and ovary derive from a common primordium when they first arise in embryos. In the mouse embryo around 9.5 dpc, cells in the adrenogonadal primordium start to express the orphan nuclear receptor *Sf1 *[also known as Nr5a1, Ad4BP, or Ftzf1 (OMIM 184757)] [12]. Between 10-11 dpc, the adrenogonadal primordium divides into adrenal primordium and gonadal primordium [13,14]. The SF1-positive cells eventually differentiate into the cortical cells of the adrenal, Sertoli and Leydig cells of the testis, and granulosa and theca cells of the ovary.

The shared origin of SF1-positive cells in adrenal and gonads raise the possibility that a common regulatory mechanism is present for the establishment or maintenance of these cell lineages. Importance of *Dicer1 *and miRNAs has been documented in the adult testis and ovary [8-11]. In this study, we developed a mouse model in which *Dicer1 *gene was inactivated specifically in the SF1-positive cells in the adrenogonadal primordium, allowing us to study the overall functions of miRNAs in the development of adrenal, testis and ovary.

## Results

### Ablation of *Dicer1 *in SF1-positive cells causes prenatal degeneration of the adrenal cortex

To investigate the functions of *Dicer1 *in development of adrenals and gonads, we generated a conditional KO model in which *Dicer1 *alleles were inactivated specifically in the SF1-positive cells, the precursors for cortical cells in the adrenals and somatic cells in the gonads [15]. The *Dicer1*-floxed allele has been shown to be a null allele upon Cre recombination in lung, limb, inner ear and germ cells [5,6,9,16]. The *Sf1/Cre *mouse line expresses high levels of Cre recombinase in the adrenogonadal primordium at 10 dpc [15]. We and others have used this *Sf1/Cre *line to remove or activate genes in the adrenogonadal primordium and observed adrenal and gonadal phenotypes at as early as 12.5 dpc [17-19].

**Figure 1:**
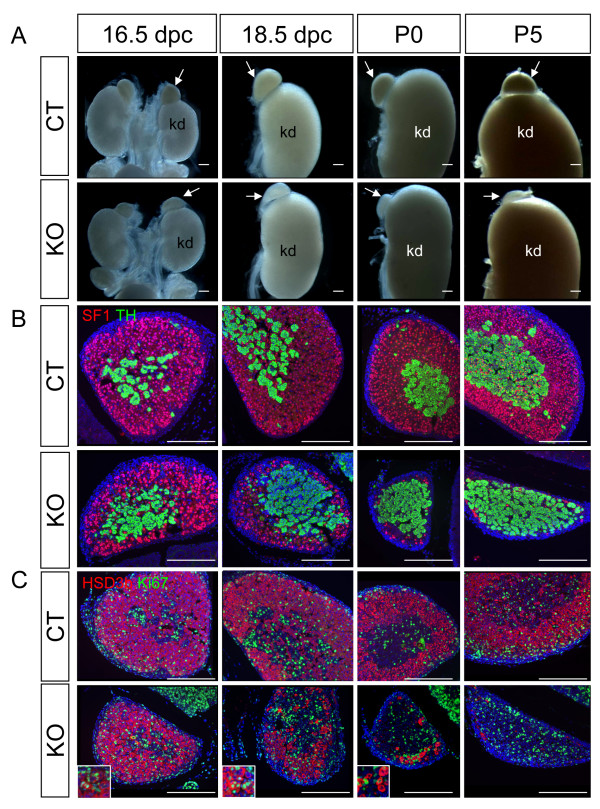
**Effects of *Dicer1 *ablation on adrenal development**. Adrenal glands from control (CT) or *Sf1/Cre;Dicer1^loxP/loxP ^*(KO) embryos (16.5 and 18.5 dpc), newborns (P0), and Day 5 neonates (P5) were collected for (A) gross morphological analysis, (B) immunofluorescence for SF1 (magenta), TH (green), and DAPI (blue), and (C) immunofluorescence for Ki-67 (green), HSD3b (magenta), and DAPI (blue). Higher magnification (2× of the original figure) of the proliferating cells is shown in the inlets. Arrow = adrenal; kd = kidney. Scale bars represent 250 μm.

Among the three organs (adrenal, testis, and ovary) that derive from the SF1-positive adrenogonadal primordium, the adrenal was the first to show histological/morphological phenotypes in response to the loss of *Dicer1*. The *Dicer1 *conditional knockout (or KO, *Sf1/Cre; Dicer1 ^floxed/floxed^*) adrenals were indistinguishable from the control adrenal (or CT, *Dicer1 ^floxed/floxed ^*or *Dicer1 ^floxed/+^*) up to 16.5 dpc. At 18.5 dpc, the *Dicer1 *KO adrenals were significantly smaller than the control and the decrease in size continued thereafter (Figure 1A). At P5, the size of KO adrenals was only 20%~30% of the control (Figure 1A). The decreased adrenal size was not the result of general growth retardation based on the fact that the body weights of control and KO animals were not different (CT, 4.02 ± 0.9 g, n = 27; KO, 3.52 ± 0.9 g, n = 6; p = 0.2). Immunofluorescence for markers of adrenal cortex (SF1) and medulla (tyrosine hydroxylase or TH) showed that the differentiation of cortex and medulla occurred properly in the KO adrenal compared to the control at 16.5 dpc (Figure 1B). However, as the adrenal development progressed, SF1-positive cells in the cortex were decreased in numbers and were almost completely lost at P5 (Figure 1B). Growth of the medulla was not affected in the KO adrenal over time, but loss of cortical cells resulted in direct contact between the medulla and the adrenal capsule in P5 KO mice (Figure 1B and Additional File 1).

**Figure 2:**
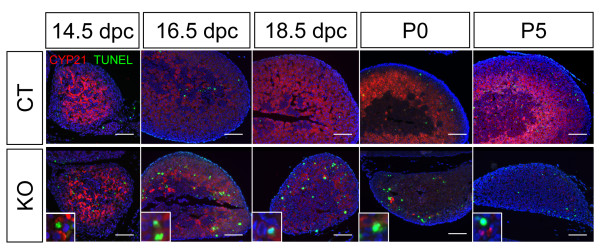
**Effects of *Dicer1 *ablation on adrenal apoptosis**. TUNEL assay was performed on sections of adrenals at 14.5 dpc, 16.5 dpc, 18.5 dpc, P0, and P5. Green nuclear staining represents positive signals for fragmented DNA and blue staining was the DAPI nuclear counterstain. Immunofluorescence for CYP21 (magenta) was also performed on adrenal sections to label the adrenal cortex. Higher magnification (4× of the original figure) of the apoptotic cells is shown in the inlets. Scale bars represent 100 μm.

To further examine the functions of the diminishing KO adrenal cortex, we analyzed the expression of the steroidogenic enzyme 3β-hydroxysteroid dehydrogenase (HSD3b) by immunofluorescence. Similar to the results of SF1 immunohistochemistry (Figure 1B), the numbers of steroidogenic cortical cells were decreased at 18.5 dpc and these cells almost completely disappeared by P5 (Figure 1C). To investigate whether the loss of cortical cells resulted from increased cell death or reduced cell proliferation, we examined proliferation by staining for proliferation marker Ki67 and apoptosis by TUNEL assay. We found no apparent changes in the number of Ki67-positive cells in the KO cortex compared to the control (Figure 1C). Using the TUNEL assay to evaluate apoptosis, we observed an increase of TUNEL- and steroidogenic enzyme 21-hydroxylase (CYP21)-double positive cells in the KO adrenal cortex starting at 14.5 dpc (Figure 2). In the control adrenal, only few TUNEL-positive cells in the cortex were observed during development (0-2 cells per section). However in the KO cortex, significant increase in TUNEL-positive cells was observed at all time points (Figure 2, average of 25 cells per section). At P5, few apoptotic cells remained in the KO adrenal where almost all cortical cells were lost at this stage. As a result of loss of adrenal cortex, none of the *Dicer1 *KO animals survived beyond P5.

### Ablation of *Dicer1 *in the SF1-positive cells causes testis degeneration after birth

**Figure 3:**
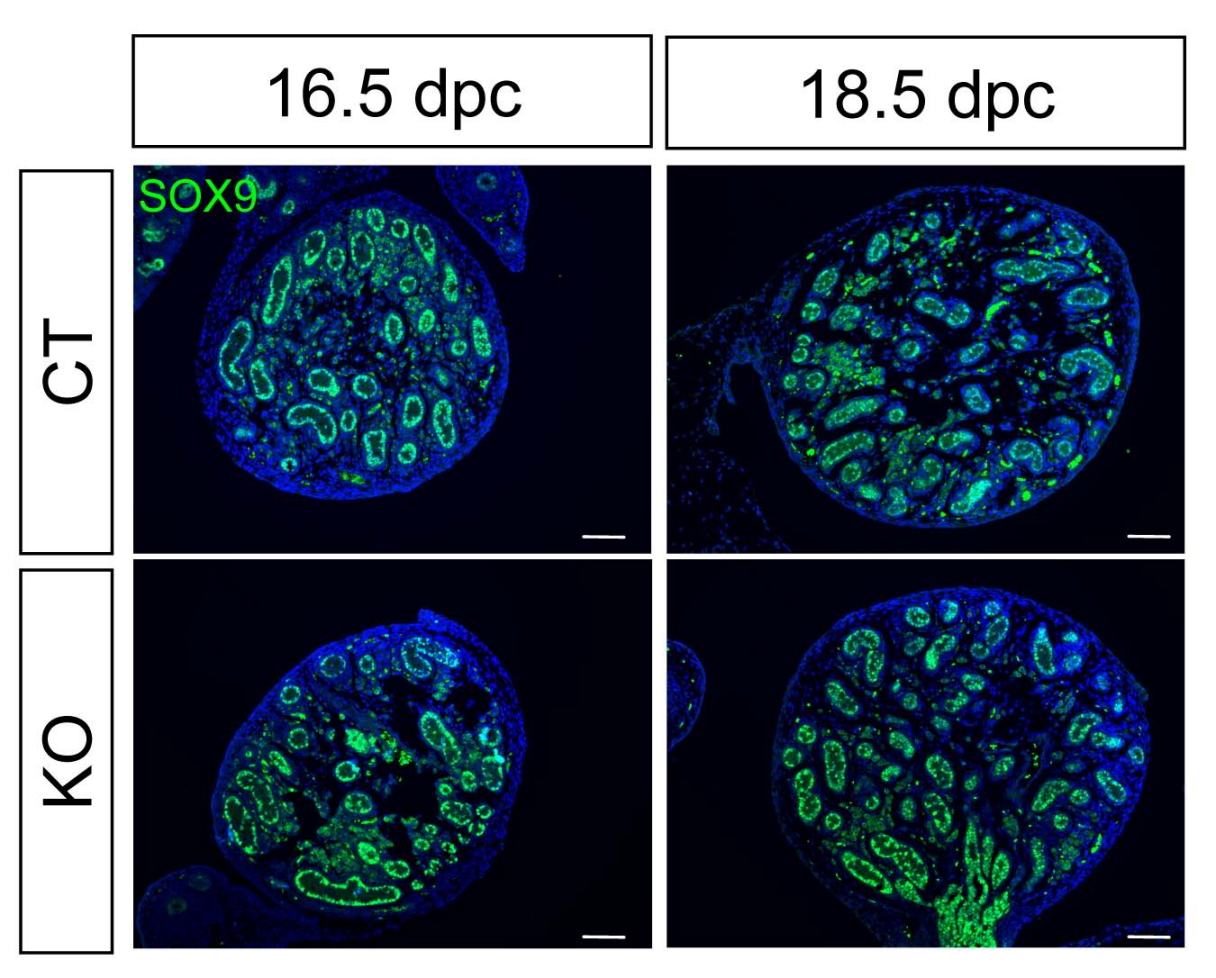
**Effects of *Dicer1 *ablation on fetal testis development**. Testes from control (CT) or *Sf1/Cre;Dicer1^loxP/loxP ^*(KO) embryos at 16.5 dpc and 18.5 dpc were collected for immunofluorescence for SOX9 (green) and DAPI counterstain (blue). Scale bars represent 100 μm.

**Figure 4:**
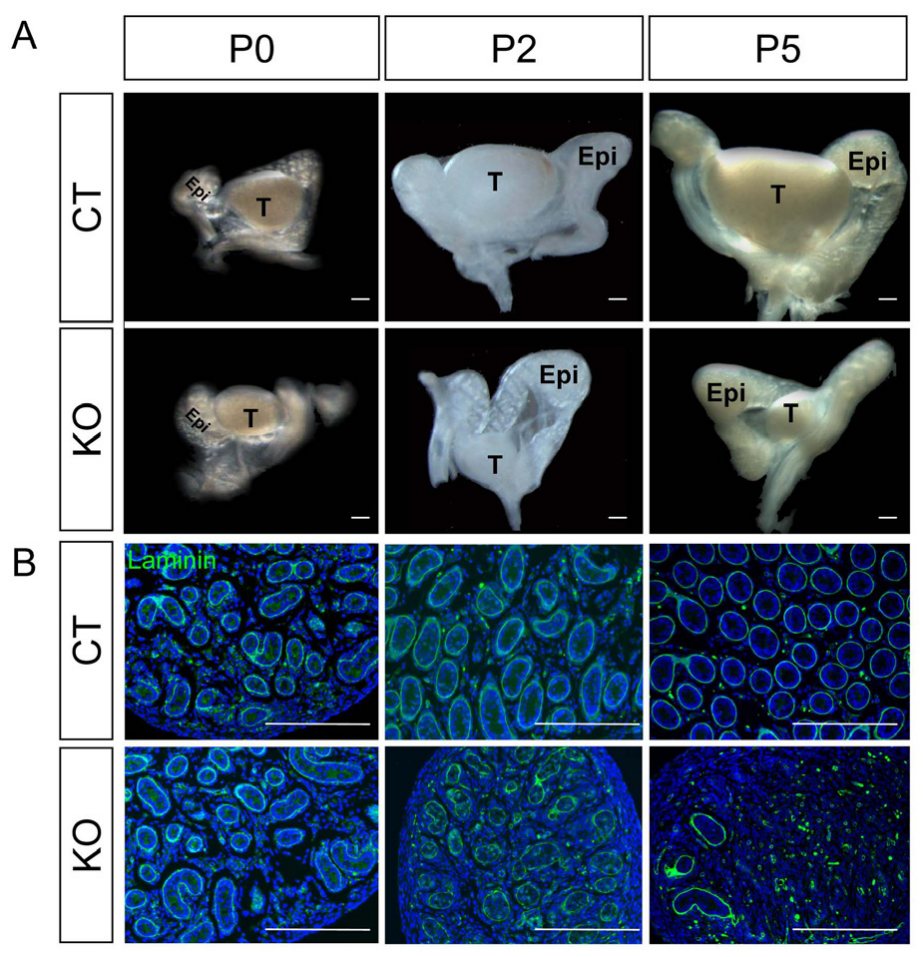
**Effects of *Dicer1 *ablation on neonatal testis development**. Testes from control (WT) or *Sf1/Cre;Dicer1^loxP/loxP ^*(KO) newborns (P0), Day 2 (P2), and Day 5 (P5) neonates were collected for (A) Gross morphological analysis and (B) immunofluorescence for laminin (green) and DAPI counterstain (blue). Epi = epididymis; T = testis. Scale bars represent 250 μm.

Ablation of *Dicer1 *mediated by *Sf1/Cre *inactivates *Dicer1 *not only in the adrenal but also in the somatic cells of fetal testes and ovaries. Although *Sf1/Cre *was activated in the adrenogonadal primordium, the KO testis did not show any abnormality during fetal life. Sertoli cell differentiation and testis cord formation in the fetal testes were comparable between control and KO as indicated by SOX9 staining (Figure 3). At the time of birth or P0, no morphological differences were found in the KO testis and epididymis (Figure 4A and 4B). At P2 and P5, the size of the KO testis remained similar to that at P0 while the size of the control testis increased over time (Figure 4A). Testis cords, as outlined by staining for laminin, started to degenerate at P2 in the KO testis and by P5, only few testis cords were observed (Figure 4B and Additional File 1).

**Figure 5:**
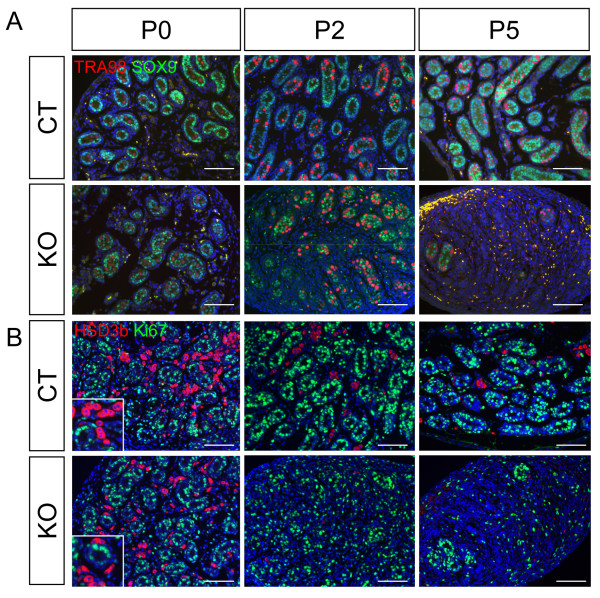
**Effects of *Dicer1 *ablation on differentiation of germ cells, Sertoli cells, and Leydig cells in the testis**. Testes from control (WT) or *Sf1/Cre;Dicer1^loxP/loxP ^*(KO) newborns (P0), Day 2 (P2), and Day 5 (P5) neonates were collected for (A) immunofluorescence for germ cell nuclear marker TRA98 (magenta), Sertoli cell marker SOX9 (green) and DAPI counterstain (blue) and (B) immunofluorescence for Leydig cell marker HSD3b (magenta), proliferation marker Ki67 (green), and DAPI (blue). Higher magnification (2× of the original figure) of the proliferating cells for P0 is shown in the inlets. Scale bars represent 100 μm.

To investigate how loss of *Dicer1 *in the SF1-positive cells affects differentiation of somatic cells and germ cells in the testis, we examined markers specific for Sertoli cells (SOX9), germ cells (TRA98) [20], steroidogenic Leydig cells (HSD3b), and the proliferation marker Ki67 (Figure 5). No significant differences were observed in KO testis at P0 compared to the control. At P2, the number of testis cords in the KO testis was decreased (Figure 5A). We also observed a decrease in the number of proliferating Sertoli cells inside the testis cords (Figure 5B and Additional File 2). At P5, KO testes lost most of the testis cord structures and few cords were present. *Dicer1 *depletion affected not only testis cords but also Leydig cells in the interstitium. At P2, most of the HSD3b-positive Leydig cells disappeared and no Leydig cells were found at P5 (Figure 5B). It is known that Leydig cells at this stage are mitotically inactive ([21,22] and Figure 5B); therefore, the decrease in Leydig cell number was probably not the result of proliferation problems.

**Figure 6:**
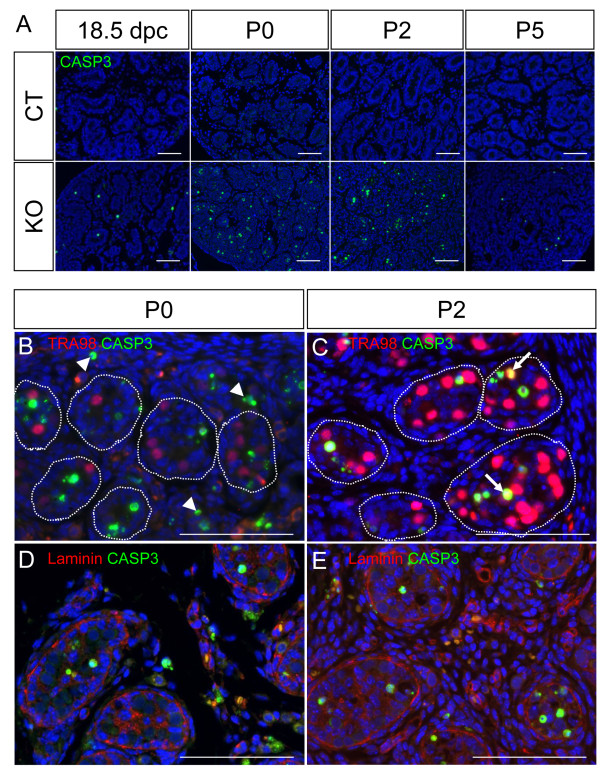
**Effects of *Dicer1 *ablation on apoptosis in the testis**. (A) Immunofluorescence for cleaved Caspase3 (CASP3) was performed on sections of testes at 18.5 dpc, P0, P2, and P5. Green nuclear staining represented positive signals for apoptotic cells and blue staining was the DAPI nuclear counterstain. Double staining of CASP3 (green) and TRA98 or laminin (magenta) was performed on testes from P0 (B and D) and P2 (C and E) KO testes. Dotted lines mark the testis cords. Arrowhead = apoptotic cells outside the testis cord. Arrow = apoptotic germ cells. Scale bars represent 100 μm.

To examine whether the degeneration of the KO testis results from increased cell death, we performed immunofluorescence for cleaved caspase-3 (CASP3), a marker for apoptosis. No CASP3-positive cells were found in the control testes during development (Figure 6A). In contrast, the KO testes had significant numbers of CASP3-positive cells starting at 18.5 dpc and becoming prominent at P0 and P2 (Figure 6A). By double staining with the germ cell marker TRA98 or the basement membrane marker laminin, we found that most of the CASP3-positive cells in P0 KO testes belonged to TRA98-negative Sertoli cells inside the testis cords and cells in the interstitium (Figure 6B and 6D). At P2, CASP3 staining was observed in both TRA98-positive germ cells and TRA98-negative Sertoli cells inside the testis cords (Figure 6C) and only few CASP3-positive cells were found in the interstitium (Figure 6E).

### Ablation of *Dicer1 *does not disturb fetal and neonatal development of the ovary

**Figure 7:**
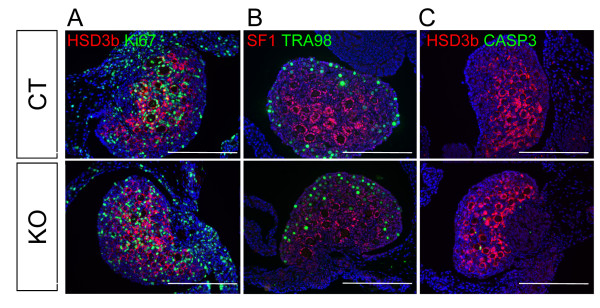
**Effects of *Dicer1 *ablation on ovary development**. Ovaries from control (CT) or *Sf1/Cre;Dicer1^loxP/loxP ^*(KO) Day 5 neonate (P5) were collected for (A) immunofluorescence for Ki67 (green) and HSD3b (magenta), (B) immunofluorescence for TRA98 (green) and SF1 (magenta), (C) immunofluorescence for CASP3 (green) and HSD3b (magenta). All sections were counterstained with DAPI (blue). Scale bars represent 250 μm.

Targeted gene deletion mediated by *Sf1/Cre *transgene occurs in all SF1-positive tissues, including the ovary [15]. In our lab, the aforementioned *Sf1/Cre *transgenic mouse line was used successfully to inactivate beta-catenin in the fetal ovary [17]. We analyzed the *Dicer1 *KO ovary mediated by the same *Sf1/Cr*e at fetal stages and up to P5 and found no differences in morphology and marker expression between control and KO ovaries (Figure 7 and Additional File 1, only P5 results are shown as representation). At P5, the size, staining patterns of cell specific markers (HSD3b and SF1 for somatic cells and TRA98 for germ cells), and markers for proliferation (Ki67) and apoptosis (CASP3) were indistinguishable between WT and KO ovaries (Figure 7).

## Discussion

In the *Sf1/Cre*-mediated *Dicer1 *KO mice, adrenal cortical cells are the first SF1-positive population that undergoes apoptosis at fetal stages, followed by testicular Leydig cells and Sertoli cells, respectively. Adrenal degeneration in the *Dicer1 *KO starts between 16.5-18.5 dpc and by the time of birth, adrenal cortical cells are almost completely abolished. In the testis, however, degeneration becomes apparent only after birth and progresses rapidly. Loss of somatic cells in the testes eventually leads to germ cells loss in the *Dicer1 *KO testis. By P5, almost all of the functional structures and cell types in the testis are no longer present. Intriguingly, ovaries, which derive from the same SF1-positive primordium as adrenal and testis, show no morphological and cellular changes from fetal stages to P5 in response to the loss of *Dicer1*. Increased apoptosis has been reported in tissues that lack *Dicer1 *[5-8]. Increased cell death in testes and adrenals in our study further suggests that *Dicer*1 and microRNAs processed by DICER play a universal role in maintaining cell survival.

In the adrenal, the cortical cells are derived from at least two sources: 1) the SF1-positive adrenal primordium, which forms the foundation of the organ and 2) SF1-negative capsular cells, which contribute to further growth of the adrenocortex [23-26]. It is known that loss of *Sf1 *leads to apoptosis of adrenal cortical cells and adrenal dysgenesis at birth [14,27]. Loss of *Dicer1 *in the SF1-positive cortical cells also leads to apoptosis of the cortex; however, the degeneration process occurs much later than that in the case of *Sf1 *KO. It is possible that DICER-regulated miRNAs control genes that are critical for cortical cells survival, such as *Sf1*. However, in the *Dicer1 *KO adrenal, SF1 is still present in the remaining cortical cells. Depletion or mutation of *Cited2, Wt1*, and *Pbx1 *also resulted in prenatal adrenal dysgenesis in mouse embryos [28-30]. Involvement of Dicer and miRNAs in regulation of these genes in adrenal development remains to be determined.

Adrenocortical degeneration was also reported in β-catenin conditional KO mice [18]. β-catenin deficiency mediated by the same *Sf1/Cre *resulted in underdeveloped adrenal cortex; however, no apoptosis was found at fetal stages. These data suggest that regulation of adrenal development via β-catenin is probably independent from DICER-regulated miRNA machinery or vice versa.

Dicer and other components of the miRNA-mediated interference machinery are present in the testis, including germ cells and Sertoli cells [31-34]. Germ cell-specific *Dicer1 *KO mice were generated using the *TNAP/Cre *that is active in the primordial germ cells [9,10]. Loss of *Dicer1 *in germ cells led to defects in proliferation and differentiation of spermatogonia and abnormal morphology and motility of sperm [9,10]. Sertoli cell-specific *Dicer1 *KO mice was generated using the anti-Müllerian hormone Cre (Amh-cre) line that targets Sertoli cells starting at ~15.5 dpc [8]. Loss of *Dicer1 *in Sertoli cells resulted in impaired spermatogenic waves and complete absence of spermatozoa. In addition, increased Sertoli cell apoptosis was found at P5 and germ cells underwent apoptosis at P15, eventually leading to complete testis degeneration at P180. In our *Sf1/Cre*-mediated *Dicer1 *KO model, we targeted both Sertoli cells and Leydig cells at an earlier stage (10-11 dpc). We observed testicular degeneration at an earlier time point and in a more dramatic fashion than was reported in the Sertoli cell *Dicer1 *KO. In our model, almost all testicular structures and cell types were absent at P5. As expected, both Sertoli and Leydig cell populations underwent apoptosis albeit within different time frames (the number of Leydig cells decreases first, followed by Sertoli cells). Although male germ cells are negative for SF1, their survival was affected in the somatic cell-specific *Dicer1 *KO testis. This is expected as Sertoli cells are known to provide structural supports and differentiation cues to support spermatogenesis [35]. Although Leydig cells were also affected in our model, we do not believe that their demise is responsible for the germ cell loss phenotypes based on the fact that male germ cells are not known to respond to androgens, the major product of Leydig cells.

*Dicer1 *is also expressed in the female reproductive tract and the ovary, including oocytes, theca cells and granulosa cells [36-39]. Many miRNAs are synthesized in ovaries at different stages of folliculogenesis [40-42]. It has been reported that morphogenesis and function of the female reproductive tract were affected in the absence of *Dicer1 *[39]. To study the *in vivo *role of *Dicer1 *in the mouse ovary, granulosa cell specific *Dicer1 *KO mice were generated by using *Amhr2/Cre*, which is expressed in granulosa cells of preantral and antral follicles [11,43-45]. Two different *Dicer1*-floxed strains were used and both showed that loss of *Dicer1 *in granulosa cells resulted in decreased ovulation rate, trapped oocytes in luteinized follicles and increased numbers of atretic follicles [11,18,46]. Fertilized oocytes collected from granulosa cell-specific *Dicer1 *KO females had decreased ability to progress to the two-cell stage [46]. We were not able to examine the consequence of loss of *Dicer1 *on folliculogenesis due to death of *Dicer1 *KO animals after P5 as a result of adrenal cortex degeneration. However at least at P5, *Dicer1 *KO ovaries show no signs of degeneration.

## Conclusions

Based on findings from our lab and others, *Sf1/Cre*-medicated gene deletion occurs in adrenals and gonads at 10-11 dpc. In the *Sf1/Cre*-mediated *Dicer1 *KO embryos, defects in adrenals and testes did not become apparent until 18.5 dpc and P0, respectively, and the ovaries were not affected. The extended delay between deletion of *Dicer *and the appearance of phenotypes suggests that *Dicer *and/or microRNAs have a substantial half-life [8]. It also raises the possibilities that (i) these organs have different turn-over rate of *Dicer *and/or Dicer-induced microRNAs, (ii) these three organs have different thresholds of tolerance toward the loss of *Dicer1 *and microRNAs, and/or (iii) Dicer and microRNAs play tissue-specific roles among these three organs. In the Sertoli cell-specific *Dicer1 *KO testis, the significant alternations in gene expression have already occurred at the time when structural changes are not yet detectable. We are currently testing these three possibilities by performing a time course analysis of changes in *Dicer1 *mRNA, Dicer protein and microRNAs in these three tissues after *Dicer1 *ablation.

## Methods

### Generation of conditional *Dicer1 *knockout mice

Conditional *Dicer1 *KO mice were generated by crossing *Sf-1/Cre *transgenic mice [15] with *Dicer1^f/f ^*mice (*Dicer1^tm1Bdh^*, obtained from the Jackson Laboratory) [5,6,9,16]. The genetic background of these mice was mixed C57BL/6J and SV129. Female and male mice were paired together and checked for the presence of a vaginal plug the next morning. The day when the vaginal plug was detected was considered 0.5 day post coitum or dpc. Samples were collected at 14.5 dpc, 16.5 dpc, 18.5 dpc, birth (P0), postnatal day 2 and 5 (P2 and P5). The genotype was determined by polymerase chain reaction (PCR) of tail DNA [6,19]. All procedures described were reviewed and approved by the Institutional Animal Care and Use Committee at University of Illinois and were performed in accordance with the Guiding Principles for the Care and Use of Laboratory Animals. All experiments were performed on at least three animals for each genotype.

### Immunofluorescence

The specimens were fixed in 4% paraformaldehyde/phosphate-buffered saline (PBS) at 4°C overnight, and embedded in paraffin following standard procedures for sectioning. For immunofluorescence analysis, paraffin embedded sections were dewaxed and rehydrated in a series of alcohol/PBS gradient. The endogenous peroxidase activity was blocked by 3% H_2_O_2 _in methanol for 8 minutes and rinsed with PBS 3 times for 5 minutes each. Slides were pretreated in 0.1 mM citrate acid for 20 minutes in the microwave. After preincubating with 1.5% normal donkey serum in PBS for 30 minutes, sections were incubated with either anti-SF1, anti-CYP21 (1:1000, kindly provided by Dr. B-c Chung, Academia Sinica, Taiwan), anti-TH (1:1000, Millipore, Billerica, MA, USA), anti-3βHSD, anti-SOX9 (1:1000, kindly provided by Dr. K. Morohashi, National Institutes of Natural Sciences, Japan.), anti-Laminin (1:500, Sigma-Aldrich, St. Louis, MO, USA), anti-TRA98 (1: 1000 kindly provided by Dr. H. Tanaka, Osaka University, Japan), anti-cleaved Caspase-3 (1:500, Cell Signaling, Danvers, MA, USA) or anti-Ki67 antibody (1:1000, BD Biosciences, San Jose, CA, USA) in PBST containing 1.5% normal donkey serum at 4°C overnight. After rinsing with PBST, sections were incubated with secondary antibody for 30 minutes and processed for signal detection according to the manufacturer's protocol (TSA kit, PerkinElmer, Waltham, MA, USA). For double-fluorescent staining of anti-cleaved Caspase-3 with other antibodies, staining of cleaved Caspase-3 were performed first as described above, samples then were double stained for the other antibodies using different fluorescent-label secondary antibodies. For other double-fluorescent staining, tissue sections were treated with two different primary antibodies generated from different species, followed by appropriate secondary antibodies. At least three animals were examined for each genotype.

### TUNEL assay

TUNEL assay was performed on 5 μm paraffin sections using Roche's TUNEL assay kit (Roche Co., Ltd., Indianapolis, IN, USA) according to the manufacturer's instructions.

## List of Abbreviations

Amh: anti-Müllerian hormone; CT: control; CYP21: 21-hydroxylase; HSD3b: 3β-hydroxysteroid dehydrogenase; KO: Knockout; PCR: polymerase chain reaction; PBS: phosphate-buffered saline; PBST: Phosphate Buffered Saline Tweeen-20; Sf1: Steroidogenic factor 1; TH: tyrosine hydroxylase; WT: wild type.

## Authors' contributions

CJH carried out all experimental works. CJH and HHY designed the experiments. CJH drafted the manuscript, which was edited by HHY. All authors have read and approved the final manuscript.

## Supplementary Material

Additional file 1 **H&E staining of adrenals, testes and ovaries of wild type and knockout mice**. Adrenals, testes and ovaries from control (CT) or *Sf1/Cre;Dicer1^loxP/loxP ^*(KO) postnatal day 5 (P5) neonates were processed for H&E staining. Images in the inlets were higher magnification. Scale bars represent 100 μm. Click here for file

Additional file 2 **Proliferating cells in the testis cords of wild type and knockout mice**. Testes from P2 and P5 control (CT) or *Sf1/Cre;Dicer1^loxP/loxP ^*(KO) neonates were examined by immunofluorescence for Ki67 (green) and SOX9 (magenta). Scale bars represent 100 μm. Click here for file
