# Supplementary figures and images for: Inactivation of Dicer1 in Steroidogenic factor 1-positive cells reveals tissue-specific requirement for Dicer1 in adrenal, testis, and ovary
Source: BMC Dev Biol. 2010 Jun 11;10:66. doi: 10.1186/1471-213X-10-66 (PMC2897782; doi:10.1186/1471-213X-10-66)

Additional File 1

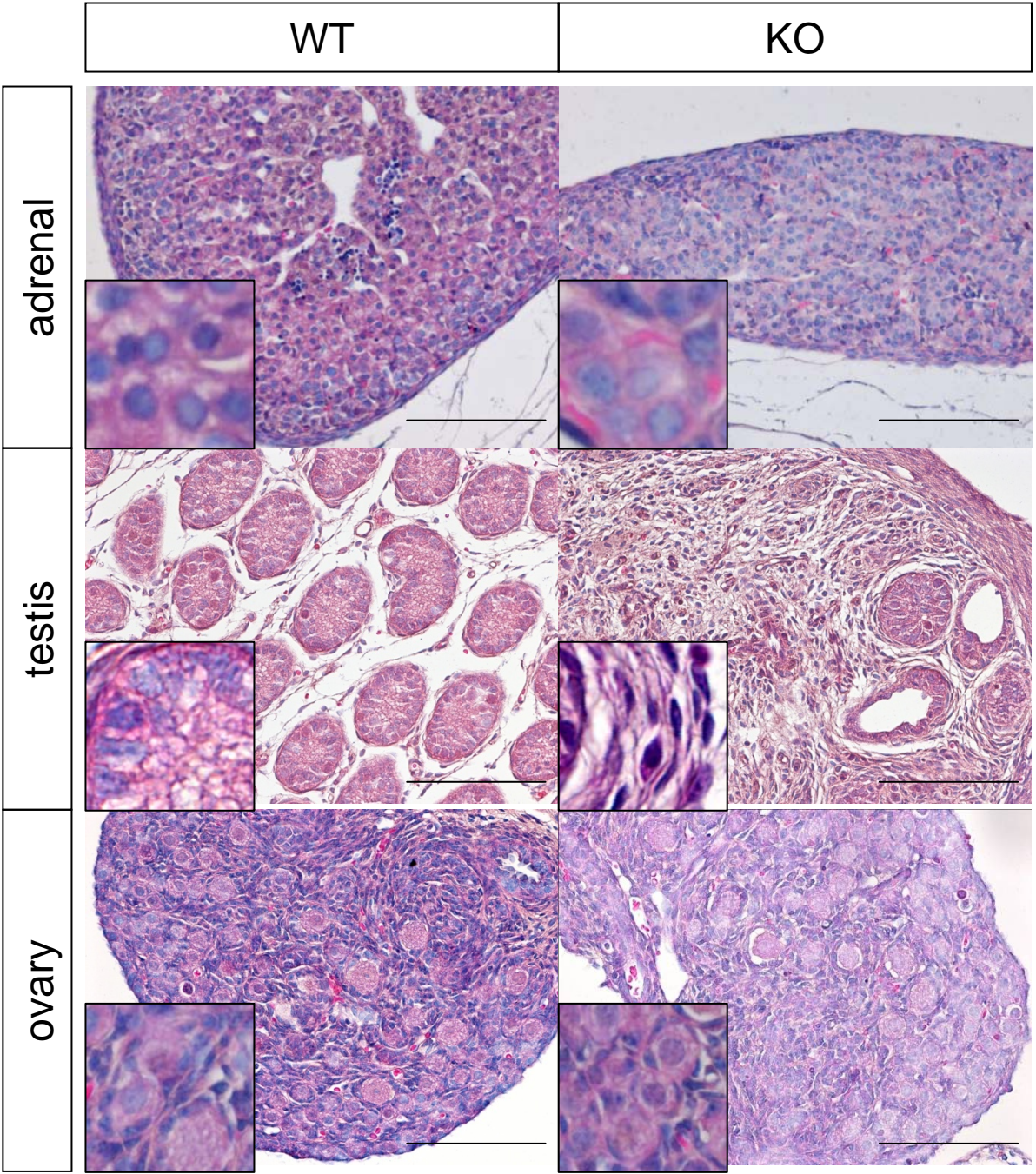

Supplement: Additional file 1 — H&E staining of adrenals, testes and ovaries of wild type and knockout mice. Adrenals, testes and ovaries from control (CT) or Sf1/Cre;Dicer1loxP/loxP (KO) postnatal day 5 (P5) neonates were processed for H&E staining. Images in the inlets were higher magnification. Scale bars represent 100 μm. [file 1471-213X-10-66-S1.PDF]

Additional File 2

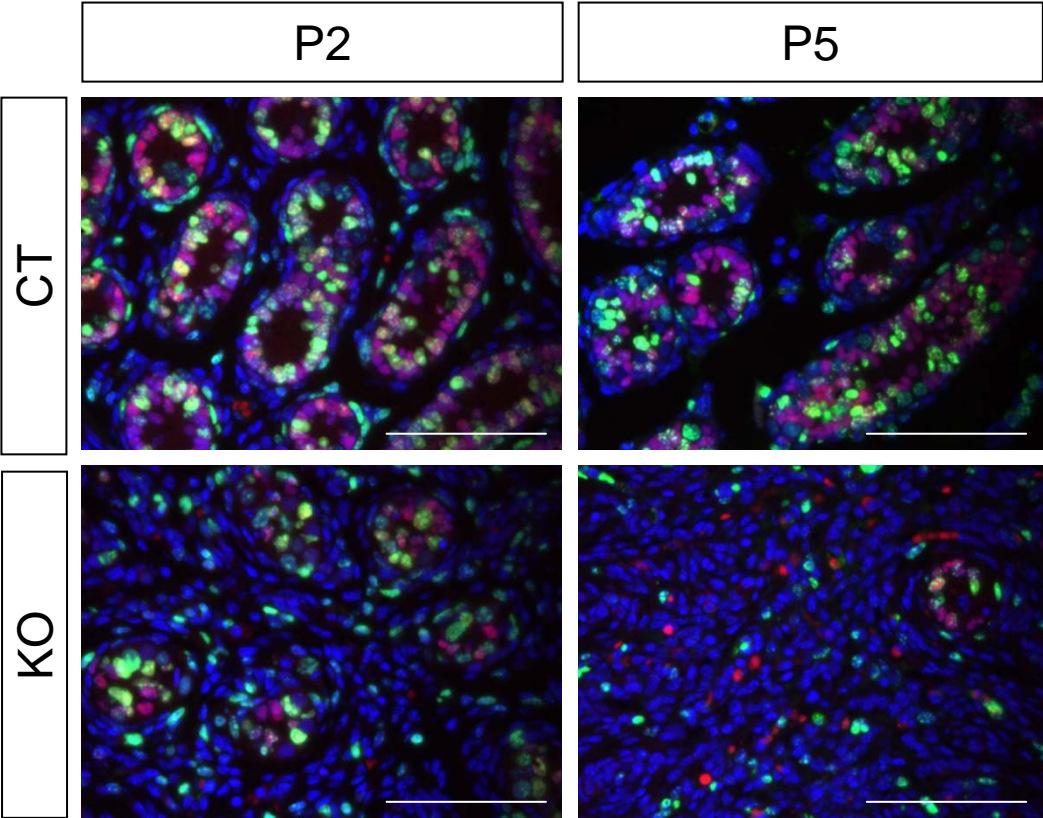

Supplement: Additional file 2 — Proliferating cells in the testis cords of wild type and knockout mice. Testes from P2 and P5 control (CT) or Sf1/Cre;Dicer1loxP/loxP (KO) neonates were examined by immunofluorescence for Ki67 (green) and SOX9 (magenta). Scale bars represent 100 μm. [file 1471-213X-10-66-S2.PDF]
